# Reduction–oxidation pathways involved in cancer development: a systematic review of literature reviews

**DOI:** 10.18632/oncotarget.17128

**Published:** 2017-04-16

**Authors:** Xīn Gào, Ben Schöttker

**Affiliations:** ^1^ Division of Clinical Epidemiology and Aging Research, German Cancer Research Center, Heidelberg, Germany; ^2^ Network Aging Research, University of Heidelberg, Heidelberg, Germany; ^3^ Institute of Health Care and Social Sciences, FOM University, Essen, Germany

**Keywords:** neoplasm, oxidative stress, redox signaling, signal transduction, reactive oxygen species

## Abstract

Oxidative stress results from an imbalance of the reactive oxygen species/reactive nitrogen species (ROS/RNS) production and the oxidants defense system. Extensive research during the last decades has revealed that oxidative stress can mediate cancer initiation and development by leading not only to molecular damage but also to a disruption of reduction–oxidation (redox) signaling. In order to provide a global overview of the redox signaling pathways, which play a role in cancer formation, we conducted a systematic literature search in PubMed and ISI Web of Science and identified 185 relevant reviews published in the last 10 years. The 20 most frequently described pathways were selected to be presented in this systematic review and could be categorized into 3 groups: Intracellular ROS/RNS generating organelles and enzymes, signal transduction cascades kinases/phosphatases and transcription factors. Intracellular ROS/RNS generation organelles are mitochondria, endoplasmic reticulum and peroxisomes. Enzymes, including NOX, COX, LOX and NOS, are the most prominent enzymes generating ROS/RNS. ROS/RNS act as redox messengers of transmembrane receptors and trigger the activation or inhibition of signal transduction kinases/phosphatases, such as the family members of protein tyrosine kinases and protein tyrosine phosphatases. Furthermore, these reactions activate downstream signaling pathways including protein kinase of the MAPK cascade, PI3K and PKC. The kinases and phosphatases regulate the phosphorylation status of transcription factors including APE1/Ref-1, HIF-1α, AP-1, Nrf2, NF-κB, p53, FOXO, STAT, and β-catenin. Finally, we briefly discuss cancer prevention and treatment opportunities, which address redox pathways and further research needs.

## INTRODUCTION

Reactive oxygen species (ROS) and reactive nitrogen species (RNS) are a battery of radical and non-radical molecules that can be produced by cellular metabolism or be induced by exogenous sources. The oxidative modification of crucial cysteine residues can lead to functional alterations of proteins, which may also have beneficial effects [1]. Therefore, ROS/RNS, especially hydrogen peroxide (H_2_O_2_) and nitric oxide (NO), can act as second messengers by activating or inhibiting protein functions [2–4]. There are various cellular activities that can be regulated by ROS/RNS via induction of redox-sensitive signal transductions [5, 6]. For instance, ROS play a part in antioxidant defense systems to maintain redox homeostasis even though they are strong oxidants themselves [3].

The term “oxidative stress” refers to an imbalance in which pro-oxidants overwhelm the capacity of antioxidant defense systems. Excessive levels of ROS/RNS are able to react with proteins, lipids and nucleic acids, and may exert negative effects on these molecules [3, 7]. Accumulating evidence has revealed that excessive levels of ROS/RNS can directly react with nucleic acids leading to mitochondrial and nucleus genomic instability, which facilitates the cancer process which in turn facilitates cancer [8]. Besides molecular damage, increased ROS/RNS levels are involved in disruption of redox signaling and regulation [9]. In cancer biology, oxidative stress has been shown to underlay the hallmarks of cancer [10]. Oxidative stress induced disruption of signaling pathways may promote cancer cell survival, proliferation, invasion, angiogenesis, inhibition of apoptosis and chemo- and radio- therapy resistance [9, 11]. Moreover, several population-based prospective studies observed that oxidative stress is a risk factor of some cancer sites [12, 13]. Therefore, there is no doubt that redox signaling pathways provide opportunities to identify novel targets of cancer treatment and prevention [14]. The present systematic review identified, appraised and summarized published literature reviews from the last ten years in order to give a comprehensive overview of the redox regulation pathways involved in cancer etiology.

## RESULTS

### Literature search and selection process

**Figure 1:**
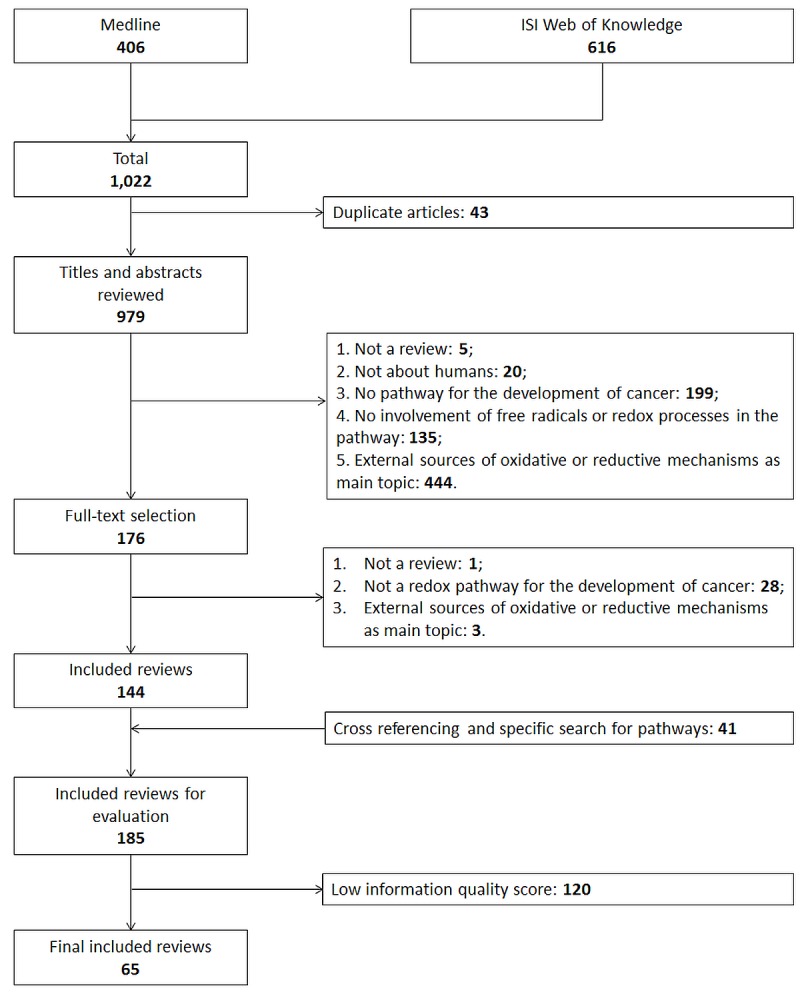
Flow chart of the systematic literature search

A flowchart of the literature search and study selection process is shown in Figure 1. 1,022 articles were retrieved by application of the search strategy to Medline and ISI Web of Knowledge and 979 articles remained after exclusion of duplicate articles. In title and abstract as well as full-text screening, articles that met 1 of the exclusion criteria were excluded and 144 articles remained. Via cross-referencing and a specific search for each pathway, we identified 38 additional reviews leading to 185 articles eligible for inclusion in this review. The 38 pathways which were related to oxidative stress and have also been described to be involved in cancer development were mentioned by the 185 relevant articles. 20 of the 38 pathways were mentioned 5 times or more and rated as the prominent pathways. In order to base this review on high-quality reviews only, information quality of the 185 review articles was scored and 120 articles were excluded with scores lower than 6 points (Appendix B) leading to the final number of 65 reviews used to describe the prominent pathways in this review (Appendix C). Each of the 20 prominent pathways was mentioned in at least in 1 high-quality review. The 20 prominent pathways can be generally categorized into 3 groups: intracellular ROS/RNS generating organelles, intracellular ROS/RNS generating enzymes, signal transduction cascades kinases/phosphatases and transcription factors (Table 1).

**Table 1:** Reduction–oxidation pathways involved in cancer development with frequency of appearance in reviews published 2005 - 2015

No	Name of pathway	Frequency of appearance in 185 included reviews	Frequency of appearance in 65 reviews with high information quality score	Selected references
Intracellular ROS/RNS generating organelles and enzymes				
1	Mitochondria	22	4	[5, 15–17]
2	Endoplasmic reticulum	3	1	[5]
3	Peroxisomes	1	1	[5]
4	NADPH oxidase	22	3	[18–20]
5	Cyclooxygenase	6	3	[21–23]
6	Lipoxygenase	6	3	[21, 22, 24]
7	Nitric oxide synthase	6	3	[25–27]
8	Cytochrome P450	1	1	[5]
9	Xanthine oxidase and oxidoreductase	1	1	[5]
10	Proline dehydrogenase	1	1	[28]
Signal transduction cascades kinases/ phosphatases				
11	Mitogen-activated protein kinase	41	6	[29–34]
12	Phosphoinositide 3-kinase	26	4	[35–37]
13	Protein tyrosine kinase	10	4	[37–40]
14	protein tyrosine phosphatase	16	4	[37, 40–42]
15	Protein kinase C	7	3	[37, 43, 44]
16	Protein kinase Hippo	2	1	[45]
Transcription factors				
17	Nrf2	29	4	[46–49]
18	NF-κB	27	4	[50–53]
19	HIF	22	4	[54–57]
20	P53	22	6	[50, 58–62]
21	AP-1	21	4	[37, 63–65]
22	FOXO	16	4	[62, 66–68]
23	STAT	10	2	[69, 70]
24	Wnt/β-catenin	7	4	[58, 71–73]
25	APE1/Ref-1	6	2	[74, 75]
26	Smad	5	2	[58, 76]
27	Yap1	4	1	[6]
28	Sp1	4	1	[6]
29	AhR	2	1	[6]
30	Ets-1	3	1	[16]
31	Egr-1	1	1	[6]
32	Glucocorticoid receptor	1	1	[6]
33	Paired box 5	1	1	[6]
34	Paired box 8	1	1	[6]
35	TTF-1	1	1	[6]
36	USF1	1	1	[6]
37	NFAT	1	1	[37]
38	Myb	1	1	[37]

Abbreviations: AhR, Aryl hydrocarbon receptor; AP-1, Activator protein 1; APE1, Apurinic/apyrimidinic endonuclease, Ref-1, Redox effector factor-1; Egr-1, Early growth response protein 1; Ets-1, E26 transformation-specific-1; FOXO, Forkhead box O; HIF, Hypoxia-inducible factor; Myb, Transcriptional activator Myb; NFAT, Nuclear factor of activated T-cells; NF-κB, Nuclear factor-κB; Nrf2, Nuclear factor E2-related factor 2; P53, Tumor protein p53; Smad, Small mothers against decapentaplegic; Sp1, Specificity protein 1; STAT, Signal transducer and activator of transcription; TTF-1, Transcription termination factor 1; USF1, Upstream stimulatory factor 1; Yap1, Yes-associated protein 1.

### Intracellular ROS/RNS generating organelles and enzymes

**Figure 2:**
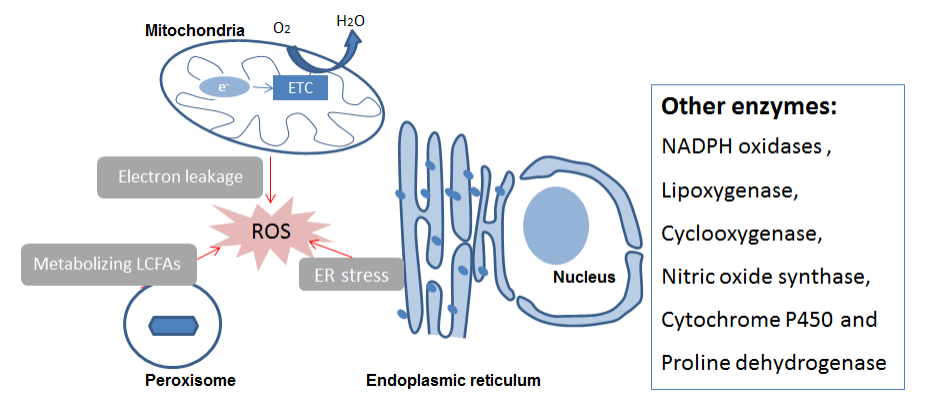
Intracellular ROS/RNS sources Abbreviations: ER, endoplasmic reticulum; ETC, Electronic transport chain; LCFAs, Long-chain fatty acids, ROS Reactive Oxygen Species.

Oxidants can be produced by organelles as well as enzyme activities. Organelles that can generate ROS are mitochondria, endoplasmic reticulum and peroxisomes. Intracellular oxidase and oxygenase can also generate ROS/RNS (Figure 2). The mechanisms indicated in Figure 2 are described briefly in the following chapters.

### Mitochondria

Mitochondria are critical for ATP biosynthesis through oxidative phosphorylation in normal cells. ROS are generated as a consequence of mitochondrial proton and electron leak which occurs in the electron transport chain (ETC) [5, 16]. The ETC consists of 5 protein complexes among which NADH-ubiquinone oxidoreductase (complex I) and ubiquinone-cytochrome c oxidoreductase (complex III) are the most investigated ROS-generating sites. Electrons are transferred from a donor molecule to an acceptor molecule during which electrons escape from ETC and are received by oxygen. Thus, oxygen is reduced to superoxide anion (O2-) which is then catalyzed to hydrogen peroxide (H_2_O_2_) by superoxide dismutase. Under physiological conditions H_2_O_2_ acts as a messenger that is essential to maintain cellular redox status [16].

Mitochondrial disruption has been found in a wide range of cancer types. Mitochondria in cancer cells generate more superoxide anions when compared to their normal counterparts [17]. It has been shown that cancer transformation induces aerobic glycolysis, which accelerates cancer cell adaption to hypoxia [15, 17]. The greater ROS stress may result from mitochondrial dysfunction and lead to oxidative damage to mitochondrial DNA (mtDNA) and nuclear DNA (nDNA) [15, 17]. Unrepaired lesions may cause oxidative phosphorylation of genes resulting in mutations which further result in an alteration of mitochondrial metabolism and bioenergetics [17]. Besides gene mutation, high levels of ROS leads to downstream pathway activation that makes cancer cells adapt to hypoxia and supports cancer cell survival [15, 17].

### NADPH oxidases (NOX)

NADPH oxidases (NOX) are widely expressed in tissues and anchored to the plasma membrane. The NOX family comprises 7 enzymes: NOX1-5 and dual oxidase1-2 (DUOX1-2) [18]. The isoforms can be divided into 2 categories by the types of ROS generation. NOX1-3 and NOX5 catalyze superoxide generation while DUOX1-2 and NOX4 promote H_2_O_2_ production [18]. NOX-mediated ROS production can be facilitated by numerous stimuli and regulated by the interaction between NOX and a battery of intracellular proteins [18, 20]. The stimuli of mammalian NOX can be generally categorized into 4 groups: chemical factors, physical factors, changed cellular environments and inflammatory factors [20]. The recruitment of cytoplasm and membrane associated proteins help NOX to improve its structural stability and activity [18].

It has been found that NOX isoforms are overexpressed in several types of cancer, indicating that NOX may promote cancer development. NOX family molecules could contribute to carcinogenesis by promoting oxidative stress and regulating cell signaling [18]. It has been shown that NOX activities are involved in regulation of cellular signaling pathways, such as Nrf2, p38 MAPK, JAK-STAT, PI3K-AKT and RAS-RAF-MEK-ERK [19]. For example: overexpression of NOX1 inhibits tumor suppressor p53 and activates NF- κB signaling, which may promote cancer development [18, 19].

### Cyclooxygenases (COX) and lipoxygenases (LOX)

Cyclooxygenases (COX) and lipoxygenases (LOX) mediate ω-6 polyunsaturated fatty acid metabolism during which ROS are generated. The overexpression of COX and LOX in certain cancers, such as prostate, colorectal, lung, breast and pancreatic cancers, implies a potential involvement in carcinogenesis [21–24].

COX is also known as prostaglandin-endoperoxide synthase. The COX family consists of 3 isoforms: COX-1, COX-2 and COX-3. COX-1 is constitutively active in multiple tissues, while COX-2 is generally undetectable in most tissues and can be induced by inflammatory stimuli and tumor promoters. COX-3 is regarded as an alternate splice variant of COX-1, but does not function in humans [22]. Both COX-1 and COX-2 can catalyze the two-steps synthesis of prostaglandin H2 from arachidonic acid. During this process arachidonic acid is oxidized to prostaglandin G2 which is then reduced to prostaglandin H2 [21]. Prostaglandin H2 is the precursor of eicosanoids which function as regulators in multiple molecular events and also play a role in promoting tumor growth and suppressing tumor immunity [21, 22]. In addition, the COX pathways can produce ROS, such as lipid hydroperoxides, and consume reduced glutathione, which leads to oxidative stress [21]. The increased ROS concentration in turn activates COX-2 [23].

In terms of LOX, 6 functional enzymes have been identified in humans, namely 5-LOX, 12-LOX, 12R-LOX, 15-LOX, 15-LOX-2 and eLOX-3 [22]. Similar to COX, LOX is able to catalyze conversion of arachnoid acid to eicosanoids and promote carcinogenesis [21, 22]. For instance, 5-LOX, the most extensively studied isoenzyme, is mainly expressed in cells of myeloid origin and has been found overexpressed in tumors [24]. 5-LOX has been shown to catalyze the conversion of arachnoid acid to 5(S)-Hydroxyicosatetraenoic acid [5(S)-HPETE] which is subsequently converted to leukotriene A4 (LTA4) [24]. These eicosanoids play a part in cell proliferation and angiogenesis [22].

### Nitric oxide synthases (NOS)

Nitric oxide (NO) is a multifunctional signaling molecule mediating the p53, NF-κB, PI3K-AKT, Wnt/β-catenin and MAPK pathways [26, 27]. NO is synthesized by nitric oxide synthases (NOS), which catalyze the synthesis of NO from L-arginine, NADPH and oxygen. The enzymatic family includes 3 isoforms: neuronal NOS (nNOS), endothelial NOS (eNOS) and inducible NOS (iNOS). nNOS and eNOS are constitutively expressed in neuronal cells and vascular endothelial cells, respectively. The expression is dependent upon intracellular calcium levels explaining why they are also termed constitutive NOS (cNOS). However, it has been observed that cNOS can also be induced by immunological stimuli and have wider distribution [28]. iNOS is predominantly distributed in macrophages and neutrophils. As the name suggests, iNOS is inducible by inflammatory cytokines, endotoxin, hypoxia and oxidative stress. In general, iNOS generates more NO for longer intervals [28].

NOSs are also expressed in malignant tumors [26]. However, NO plays quite a complex role in tumors and it depends on the concentrations, timing, location and presence of other free radicals [26, 27]. In normal cells, NO derived from iNOS mediates chronic inflammation, which may promote neoplastic transformation [26]. In tumor cells, lower levels of NO can prevent some types of tumor cells from apoptosis. However, higher NO concentrations can be toxic to tumor cells [27]. For instance, in vascular endothelial cells, moderate NO derived from eNOS functions, activates matrix metalloproteinase family molecules via the RAS-RAF-MEK-ERK pathway facilitating angiogenesis [25].

### Other intracellular ROS/RNS generating sources

The endoplasmic reticulum and peroxisomes are also organelle sources of ROS/NOS, as shown in Figure 2. They are described in detail in the review of Holmstrom and Finkel [5]. In brief, the endoplasmic reticulum is a well-orchestrated protein-folding organelle and can produce ROS as by-products. ROS generation is increased during endoplasmic reticulum stress in response to accumulation of misfolded and unfolded proteins. Peroxisome is involved in various biochemical processes and it can produce ROS as part of their role in metabolizing long-chain fatty acids. For less prominent intracellular ROS/RNS generating enzymes, we refer interested readers to other reviews. Holmstrom and Finkel [5] give an overview on cytochrome P450 as well as xanthine oxidase and oxidoreductase. Another enzyme, proline dehydrogenase, is discussed in the review of Liu and Phang [28].

### Signal transduction cascades kinases/phosphatases

Generally, ROS/RNS interact with some certain amino acid residues of proteins (protein tyrosine phosphatases (PTPs), protein tyrosine kinases (PTKs) and protein kinase C (PKC) and in turn activate downstream kinase cascades (phosphoinositide 3-kinase (PI3K) and mitogen-activated protein kinases (MAPKs). They function as a switch of downstream proteins in many cellular functions and detailed mechanisms are described below.

### Mitogen-activated protein kinases (MAPKs)

**Figure 3:**
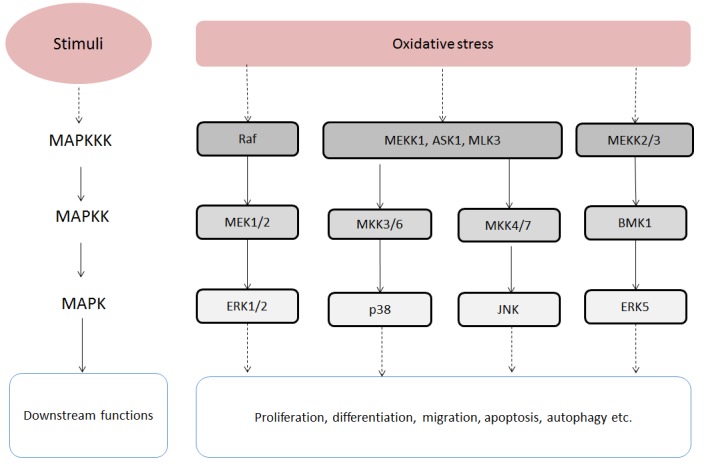
Oxidative stress-stimulated MAPK signalling pathways Abbreviations: ASK1, Apoptosis signal-regulating kinase 1; BMK1, Big mitogen-activated protein kinase 1; ERK1/2, Extracellular signal-regulated kinase 1/2; ERK5, Extracellular signal-regulated kinase 5; JNK, c-Jun N-terminal kinases; MAPK, Mitogen-activated protein kinase; MAPKK, MAPK kinase; MAPKKK, MAPK kinase kinase; MEK1, MAPK/ERK kinase 1; MEK2, MAPK/ERK kinase 2; MEKK1, MAPK/ERK kinase kinase 1; MEKK2, MAPK/ERK kinase kinase 2; MEKK3, MAPK/ERK kinase kinase 3; MKK3/6, MAPK kinase 3/6; MKK4/7, MAPK kinase 4/7; MLK3, mixed-lineage protein kinase 3; p38, P38 mitogen-activated protein kinase.

The mitogen-activated protein kinase (MAPK) signaling pathways modulate a wide range of cellular responses, such as proliferation, differentiation, migration, apoptosis and autophagy [30]. MAPKs can be activated by many stimuli and activation can have numerous consequences. Therefore, the roles of the MAPK signaling pathways in cancer development are complex and pleiotropic [31]. Typically, the MAPK signaling pathways consist of 3 sequential kinases: a MAPK kinase kinase (MAPKKK) activates a MAPK kinase (MAPKK) which subsequently activates a MAPK (Figure 3) [30]. There are 4 MAPK family members, which are activated by ROS, including Extracellular Regulated Kinases 1/2 (ERK1/2), Jun N-terminal Kinases (JNKs), P38 mitogen-activated protein kinases (p38) and Big MAPK Kinase 1 (BMK1) [29].

The ERK1/2 signaling pathway, also referred to as the RAS-RAF-MEK-ERK pathway, is an extensively studied MAPK pathway. Oxidative stress is able to induce ERK activation through the RAS-RAF-MEK-ERK kinases axis [29]. The activation of ERKs modulates several cellular activities, such as cell survival, cell proliferation, cell migration and differentiation [29, 30]. Therefore, the ERK1/2 pathway can promote cancer development by maintaining cancer cell survival and stimulating cancer cell proliferation and metastasis [30, 34]. In addition, mutations in genes which encode for RAS and RAF are involved in cancer development [34].

The JNK and p38 MAPK pathways have common upstream regulators and can be activated by oxidative stress [33]. They also interact with each other at the level of downstream targets which have been linked to cell proliferation, differentiation, survival and migration [33]. In the p38 MAPK signaling pathway, ROS/RNS can activate the p38 MAPK pathway by activating MAPK/ERK kinase kinase 1 (MEKK1), mixed-lineage protein kinase 3 (MLK3) and apoptosis signal-regulating kinase 1 (ASK1). Then the MAPKKKs in turn activate MAPK kinase 3 and MAPK kinase 6 (MAP2K3/6) [29, 30]. The activated p38 MAPK targets multiple transcription factors, such as p38, MEF2 and ATF-2 [34]. In the JNK signaling pathway, ROS can induce the activation of ASK1 and MAP3K1 which are MAPKKK. Then the MAPKKK activate MAP2K4/7 which subsequently activates JNKs. Similar to p38 MAPK, the activated JNKs are able to phosphorylate several transcription factors, like c-Jun, ATF-2 and STAT3 [29]. However, the p38 MAPK and JNK pathways produce contradictory effects on cell proliferation, apoptosis and differentiation in different cell contexts [33].

The most recently discovered MAPK pathway is the BMK1 signaling pathway which can also be induced by oxidative stress. This pathway is similar to the mentioned MAPK pathway with MEKK2/3 activating MAP2K2 and MAP2K2 subsequently activating BMK1 [29, 32]. Experimental evidence implies that the BMK1 signaling pathway is promoting angiogenesis and metastasis in cancer [32].

### Phosphoinositide 3-kinase (PI3K)/AKT

**Figure 4:**
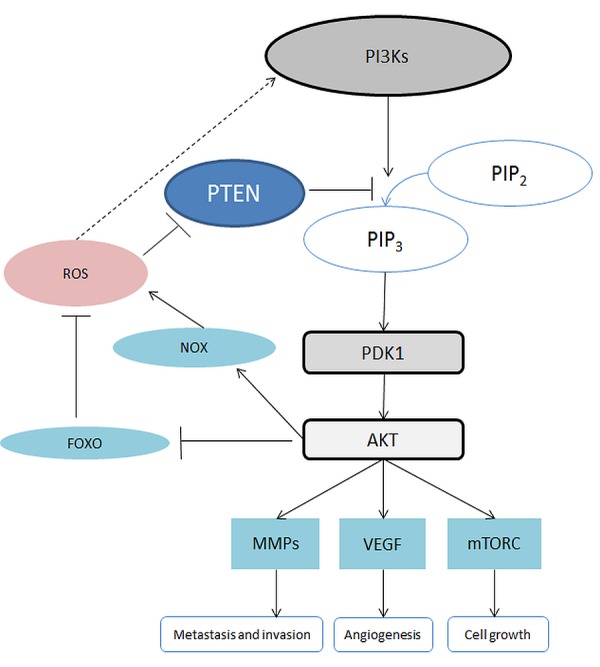
Schematic of the PI3K/AKT signalling pathway Abbreviations: AKT, also known as protein kinase B, PKB; FOXO, Forkhead Box O; MMP, Matrix metalloproteinase; mTORC, mammalian Target of Rapamycin Complex; NOX, NADPH Oxidase; PDK1: Phosphoinositide-Dependent Kinase-1; PIP2, Phosphatidylinositol 4,5-bisphosphate; PIP3, Phosphatidylinositol-3,4,5-trisphosphate; PTEN, Phosphatase and tensin homolog; ROS, Reactive Oxygen Species; VEGF, Vascular Endothelial Growth Factor.

In humans, the phosphoinositide 3-kinase (PI3K) family includes a variety of enzymes which have been divided into 3 classes: class I, class II and class III. We discuss herein class I which is the most investigated class involved in cancer development. The PI3Ks signaling pathway is triggered by growth factors and, in some cases, by ROS (Figure 4) [37]. The activated PI3Ks then phosphorylate phosphatidylinositol-4,5-bisphosphate (PIP2) to phosphatidylinositol-3,4,5-trisphosphate (PIP3) which acts as a signaling molecule to recruit pleckstrin homology domain containing proteins, such as phosphoinositide-dependent kinase-1 (PDK1) and protein kinase B (PKB, also known as AKT) [36]. The PI3K/AKT signaling pathway can be downregulated by phosphatase and tensin homolog (PTEN), which can dephosphorylate PIP3 back to PIP2 [77]. However, PTEN can be oxidized and inhibited by H_2_O_2_ resulting in activation of the PI3K/AKT signaling pathway.

The activation of AKT is involved in multiple cellular activities. For instance, AKT is involved in the activation of NOX, an enzyme generating ROS [77]. In addition, AKT mediates inhibition of forkhead box O (FOXO). The inactivation of FOXO also results in inhibition of apoptosis, which favors cancer growth [35]. AKT signaling, which has been shown to modulate many downstream pathways, is one of the major mediators of the PI3K pathway [35]. The hyperactivation of the PI3K-AKT pathway is regarded as a hallmark of cancer. The activation of the PI3K-AKT signaling pathway is engaged in the inhibition of apoptosis as well as the promotion of proliferation and angiogenesis [36]. AKT can induce stimulation of mammalian target of rapamycin complex 1 (mTORC1) which promotes synthesis of proteins and cell survival [35, 36]. In addition, AKT signaling contributes to angiogenesis and metastasis by triggering the vascular endothelial growth factor pathway (VEGF) and promoting the secretion of matrix metalloproteinase (MMP) [35]. MMP supports metastasis and invasion of cancer cells [78].

### Protein tyrosine kinases (PTKs) and protein tyrosine phosphatases (PTPs)

Protein tyrosine kinases (PTKs) have been found to be hyperactivated in many cancer cells and are regarded as oncoproteins. The PTK superfamily is composed of 2 large enzyme families: the receptor tyrosine kinases (RTK) family (with 7 subfamilies) and the cytoplasmic non-receptor tyrosine kinases (NRTK) family (with 9 subfamilies) [37–40]. RTK isoforms are anchored to the plasma membrane and behave as cell surface receptors for various signaling molecules including H_2_O_2_ [37, 40].

Integrin-mediated RTK activation is involved in regulating cancer cell division, adhesion, angiogenesis, motility and survival [39]. An example of RTK is the vascular endothelial growth factor receptor family which binds to vascular endothelial growth factor and induces vasculogenesis and angiogenesis in solid tumors [38]. NRTK isoforms are found in the cytoplasm and function as downstream targets of RTKs. They are vital components in various cancer promoting signaling pathways, such as the MAPK and PI3K pathways [39] (see also previous chapters). Through the activation of these pathways, NRTKs facilitate cancer cell transformation, proliferation and migration and inhibit apoptosis [39]. For instance the Src family kinases, which belong to the largest subfamily of NRTKs, are activated in a wide variety of cancer sites [39].

The term PTP defines a superfamily of more than 100 enzymes consisting of 2 subgroups: classical phosphotyrosine (pTyr)-specific phosphatases (classical PTPs) and dual specificity phosphatases [41]. There are several mechanisms involved in PTP regulation, including oxidation [41]. It has been found that ROS-mediated oxidation can result in inactivation of PTPs. PTPs are involved in modulation of signaling pathways in coordination with PTK [41]. It has been shown that PTPs negatively regulate PTKs [37, 40]. Therefore, ROS can indirectly activate PTKs via inactivating PTPs [37, 40]. Some PTPs behave as tumor suppressors [41]. For example, phosphatase and tension homolog, which is one of the most described dual specificity phosphatases, can mediate downregulation of the PI3K pathway resulting in tumor-cell death ([41], see also the chapter “Phosphoinositide 3-kinase (PI3K)/AKT”). There are also some PTPs that upregulate RTK signaling and function as cancer promoters [42]. One of the oncogenic PTPs is SHP2 that activates the Src family kinases by dephosphorylating a C-terminal binding site [42].

### Protein kinase C (PKC)

Protein kinase C (PKC) comprises a large family of serine/threonine kinases with 3 subfamilies, which are: conventional PKCs, novel PKCs and atypical PKCs. ROS/RNS can modulate PKCs by modifying the cysteine-rich zinc-finger regions of PKCs. H_2_O_2_ for example, activates PKC by inducing phosphorylation [44]. PKCs are not only regulated by ROS but are also related to ROS production. PKCs activation promotes the activity of NOX, which increase the level of ROS [37].

Activated PKC isozymes are also involved in many cellular signaling pathways by phosphorylating downstream proteins [44]. It has been shown that PKCs are upregulated in several types of cancer cells [43]. They are implicated in cancer cell survival, proliferation, invasion, metastasis, angiogenesis and apoptosis [43, 44]. However, whether PKCs promote or inhibit cancer development is dependent on cell context such as the isoform of PKC and the type of cancer [43]. PKCα, a member of the conventional PKC subfamily, acts as a promoter of cell survival by regulating activation of NF-κB. Another example is PKCδ which is engaged in promoting apoptosis by regulating the MAPK pathway [44].

### Other signal transduction cascades kinases/phosphatases

For the less prominent signal transduction cascade kinase protein kinase Hippo we would like to refer interested readers to the review of Qin et al. [45].

### Transcription factors

Transcription factors are proteins that regulate target gene expression. The ROS-regulated transcription factors introduced in this chapter are Nrf2, NF-κB, HIFs, P53, AP-1, FOXO, STATs, β-catenin, APE1/Ref-1 and Smad proteins. ROS/RNS regulate these transcription factors through activation of protein kinases, inactivation of phosphatases or direct redox reaction with transcription factors [6].

### Nuclear factor E2-related factor 2 (Nrf2)

**Figure 5:**
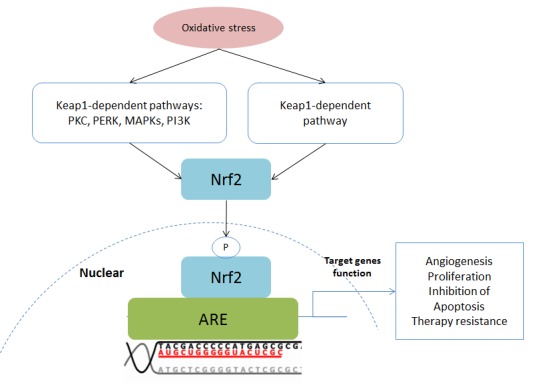
Schematic of the Keap1-Nrf2-ARE pathway Abbreviations: AKT, also known as protein kinase B, PKB; FOXO, Forkhead Box O; MMP, Matrix metalloproteinase; mTORC, mammalian Target of Rapamycin Complex; NOX, NADPH Oxidase; PDK1: Phosphoinositide-Dependent Kinase-1; PIP2, Phosphatidylinositol 4,5-bisphosphate; PIP3, Phosphatidylinositol-3,4,5-trisphosphate; PTEN, Phosphatase and tensin homolog; ROS, Reactive Oxygen Species; VEGF, Vascular Endothelial Growth Factor.

Nuclear factor E2-related factor 2 (Nrf2) is a bZIP protein and plays a central role in the Kelch-like ECH-associated protein 1 (Keap1)-Nrf2-ARE pathway (Figure 5). This pathway is involved in the regulation of antioxidant proteins that protect cells against oxidative stress [46–49]. Keap1 is an important inhibitor of this pathway. The formation of a Keap1-Nrf2 complex leads to degradation of Nrf2. However, oxidative stress or electrophilic stress results in dissociation of the Keap1-Nrf2 complex facilitating Nrf2 activation. In addition, Nrf2 can also be activated by oxidative stress via the PI3K, PKC and MAPK pathways [46]. Then Nrf2 proteins translocate into the nucleus and bind to the antioxidant response element (ARE) leading to target gene expression [46]. The Nrf2 is a master regulator of antioxidants, such as glutathione, heme oxygenase-1, catalase, superoxide dismutase and thioredoxin [46–49]. Besides antioxidants, the activation of Nrf2 results in expression of proteins which are responsible for anti-apoptosis. For instance, Bcl-2, a downstream protein of Nrf2, is an inhibitor of apoptosis [47].

However, Nrf2 has a dual role in cancer development and progression [46, 47, 49]. As mentioned previously, Nrf2 maintains intracellular redox homeostasis and prevents normal cells from conversion into cancer cells. Nrf2 is a cancer suppressor when it is activated in normal cells. However, it has been shown that Nrf2 proteins are overexpressed in cancer cells [46]. The hyperactivated Nrf2 contributes to overexpression of target genes, leading to cancer cell survival and proliferation [46–49]. In addition, Nrf2 helps cancer cells to resist chemotherapy and radiotherapy [46, 49]. Therefore, Nrf2 behaves as a cancer-promoter in cancer cells.

### Nuclear factor-κB (NF-κB)

Nuclear factor-κB (NF-κB), which are ubiquitous in mammalian tissues, regulate expression of numerous inflammatory mediators and act as core factors in multiple immune responses. This proteins family consists of 5 members: RelA (p65), RelB, c-Rel, NF-κB1 (p50/p105) and NF-κB2 (p52/p100) [50, 51, 53]. NF-κB transcription factors have the same conserved N-terminal Rel homology domain and can interact with each other forming up to 15 homo- and heterodimeric complexes [51]. In resting cells, the transcriptional function of NF-κB dimers is blocked by a set of proteins, such as inhibitor of kappa B (IκB) family proteins (IκBα, IκBβ and IκBε), p50 and p52 [51]. It has been shown that the NF-κB pathway can be stimulated by oxidative stress via activation of IκB kinases (IKKs) [51–53]. Once stimulated, the NF-κB pathway upregulates the expression of target genes in apoptosis, proliferation and immune response [51, 52]. In addition, the NF-κB pathway interacts with other redox signaling pathways, such as the JNK pathway.

NF-κB genes have been recognized as proto-oncogenes [50, 52, 53]. It has been observed that NF-κB dimers are constitutively active in several types of cancer cells, which accelerates cancer cell proliferation, invasion, metastasis, angiogenesis and inhibition of apoptosis [52]. For instance, NF-κB activation increases the expression of MMP-9 promoting metastasis and invasion [52, 53]. Activated NF-κB dimers can also upregulate the epithelial growth factor inducing proliferation [52]. In addition, it has been shown that chemotherapeutic agents and ionizing radiation stimulate NF-κB dimers activation leading to resistance against these therapies [52, 53].

### Hypoxia-inducible factors (HIFs)

Hypoxia-inducible factor (HIFs) consist of the 3 isoforms HIF-1, HIF-2 and HIF-3. Each of the HIFs has 2 subunits, α and β. HIF-1α is a transcription factor that is ubiquitous in mammalian cells and acts as an essential mediator in response to a low oxygen microenvironment [57]. In normoxia, prolyl hydroxylases hydroxylate 2 prolines on HIF-1/2α inducing ubiquitination and degradation of HIF-1/2α [57]. Hypoxia can induce inhibition of prolyl hydroxylases leading to dimerization of HIF-1/2α and HIF-1/2β. The HIF heterodimer regulates expression of target genes [57]. ROS/RNS have been shown to regulate HIF-1α activity through the PI3K and MAPK cascade pathways [55]. Increased ROS/RNS levels induce stabilization, accumulation and activation of HIF-1α even in normoxia [57]. It has been suggested that HIF-1α is overexpressed in different tumor types and is implicated in excessive vascularization and tumor invasiveness [55]. For example, HIF-1α upregulates the expression of vascular epidermal growth factors which stimulate angiogenesis in cancer [55]. HIF-2α also represents transactivation activity. However, it is expressed in more restricted cell types and functions differently when compared to HIF-1 [56]. Hypoxia in tumors leads to overexpression of HIF-2α which subsequently favors cancer cell survival, proliferation and metastasis [56]. Knowledge about HIF-3α is limited because of the diversity of HIF-3α variants [54]. It has been shown that several HIF-3α variants regulate HIF-1/2α activities negatively [54].

### Tumor protein p53

Tumor protein p53 is a vital redox-sensitive transcription factor that exerts suppressor effects on cancer development. It has been shown that oxidative stress can change the intracellular concentrations and subcellular localization of the p53 protein [50, 58, 62]. ROS cause oxidative modification of p53 cysteine residues affecting its transcriptional activity [59]. In addition, oxidative stress induces p53 activation by stimulating several signaling pathways (APE1/Ref-1, ASK1-p38 MAPK and PTPs-PKCδ pathways) [62]. Furthermore, the p53 protein is sensitive to ROS inducing DNA double-strand breaks via the ataxia telangiectasia mutated pathway [62]. It also regulates various target genes, which are engaged both in ROS/RNS generation (p53 inducible proteins family, COX-2 and NOS2) and in ROS/RNS clearance (glutathione peroxidase and superoxide dismutase) [59, 62].

The p53 protein represents defensive responses to carcinogens. However, the p53 gene has been found mutant across all cancer sites leading to dysregulation of downstream genes [58, 61]. The p53 inducible protein 3, as a prominent example, induces ROS generation in order to stimulate genotoxic stress that leads to apoptosis [61]. Most tumor-derived p53 mutants lead to an inactivation of p53 inducible protein 3, which promotes cancer cell survival [61]. Superoxide scavenger superoxide dismutase is another downstream protein of p53. It can be upregulated by physiological levels of p53 and shows an antioxidative ability to protect cells from apoptosis [60, 62]. However, high levels of p53 induce superoxide dismutase downregulation which increases ROS concentration and promotes apoptosis [60, 62]. Superoxide dismutase is found overexpressed and is in favor of mitochondrial homeostasis in cancer cells [60].

### Activator protein 1 (AP-1)

Activator protein 1 (AP-1) is also characterized by a basic leucine-zipper (bZIP) and can bind to 12-O-tetradecanoylphorbol-13-acetate (TPA) or cMAP response elements (CRE) to regulate gene expression [37, 63]. It comprises members from the FOS (c-FOS, FRA1, FRA2, FOSB), JUN (c-JUN, JUNB, JUND), ATF/CREB (CREB, ATF1-7, B-ATF, ATFa), MAF (c-MAF, MAFB, NRL, MAFF/G/K) and JDP (JDP1/2) subfamilies [63]. AP-1 is a dimeric complex. Its regulations include dimer composition, transcription, post-translational modification and interactions with other proteins [63]. It has been reported that oxidative stress is able to induce upregulation as well as downregulation of AP-1 activity by modifying AP-1 at the post-translational and post-transcriptional levels [37].

Target genes of AP-1 are involved in invasion, metastasis, proliferation, differentiation, survival and angiogenesis [63, 64]. However, AP-1 is a double-edged sword in tumorigenesis because its actions depend upon relative abundance of subfamily members [65]. It can be a pro-oncogenic or anti-oncogenic factor. A pro-oncogenic example is that c-JUN and JUNB promote uncontrolled cell division by inducing upregulation of the epidermal growth factor receptor [64, 65]. An anti-oncogenic example is that c-JUN can downregulate the expression of p53 resulting in inhibition of proliferation and stimulation of apoptosis [65].

### Forkhead box O (FOXO)

The forkhead box O (FOXO) protein family is a subfamily of forkhead transcription factors. They participate in cell fate determination. There are 4 isoforms that have been identified in mammals: FOXO1, FOXO3a, FOXO4 and FOXO6. FOXO proteins are regulated by ROS and are implicated in regulation of intracellular redox status [62, 66, 68]. It has been shown that FOXO proteins can be inhibited by ROS via the PI3K and IKK pathways [67, 68]. However, ROS can also act as an activator of the FOXO proteins by inducing activation of the MAPK pathways [67, 68]. Target genes of FOXO proteins are devoted to multiple cellular activities, such as detoxification of ROS, cell cycle arrest and apoptosis [66, 68]. At the early stage of cancer development, FOXO activity is inhibited by the activation of the PI3K-AKT pathway, which causes accumulation of ROS and drives cancer progression [67].

### Signal transducer and activator of transcription (STAT)

Signal transducer and activator of transcription (STAT) proteins are transducers and transcription factors involved in the regulation of cellular inflammatory responses, cell survival and proliferation. Seven STAT proteins have been identified in mammals: STAT1-4, STAT5A, STAT5B and STAT6 [69, 70]. STAT proteins are induced by activation of cytokine-Janus kinase (JAK) signaling [69, 70]. ROS/RNS have been shown to regulate STAT negatively or positively [69]. Oxidative stress may inhibit the cytokines and oxidize STAT and JAK resulting in inactivation of the cytokine-JAK-STAT pathway [69]. On the contrary, it has also been reported that H_2_O_2_ has the ability to activate the pathway via inhibition of tyrosine phosphatases and activation of tyrosine kinases [69]. Furthermore, intracellular redox status can be regulated by STAT proteins. For instance, STAT3 can migrate into mitochondria and upregulate ETC activity which leads to an increase in ROS production [69].

STAT proteins control inflammatory responses and are involved in cancer development [70]. STAT3 and STAT5 are upregulated in numerous types of cancer cells and promote cancer growth, angiogenesis and metastasis [70]. In particular, activation of STAT3 leads to expression of interleukin-6 and COX-2 which mediate cancer-promoting immunity [70].

### Wnt/β-catenin pathway

The Wnt/β-catenin signaling pathway is implicated in cell proliferation, cell adhesion and cell fate decision [58, 71, 73]. Under unstimulated condition, β-catenin is inactivated by forming a destruction complex with axin, adenomatous polyposis coli, glycogen synthase kinase 3 and casein kinase 1 [71, 72]. The Wnt protein, which consists of 19 isoforms, is an upstream machine in the pathway. Wnt can activate β-catenin transcriptional activity by binding to a Frizzled receptor and low-density lipoprotein receptor-related protein 5 and 6 [71, 72]. ROS have been shown to exert a positive role in regulation of the Wnt/β-catenin pathway through inhibiting the activity of a blocker of the Wnt/β-catenin pathway, named nucleoredoxin. ROS are also involved in interactions between the Wnt/β-catenin and downstream signaling pathways, such as p53 and FOXO [58]. It has been shown that the misregulation of the Wnt/β-catenin pathway is associated with cancer development. Activating mutations of the Wnt/β-catenin pathway components have been found in many types of cancer (e.g. hepatocellular cancer [73]), which benefits cancer growth [71–73]. For example, mutations in the β-catenin gene (CTNNB1) can lead to β-catenin overexpression.

### Apurinic/apyrimidinic endonuclease 1 (APE1)/Redox effector factor-1 (Ref-1)

**Figure 6:**
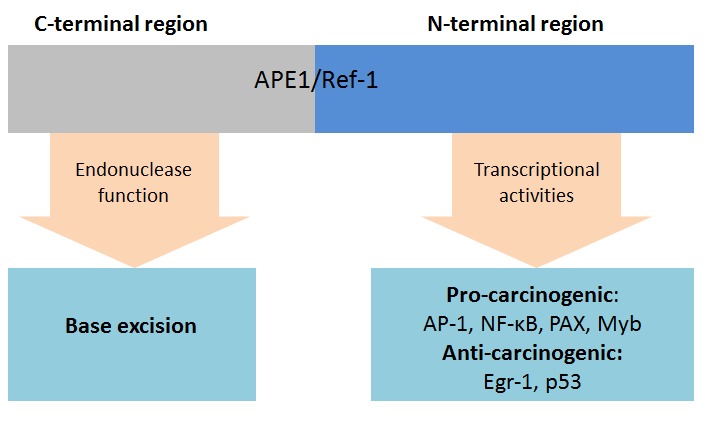
Functions of APE1/Ref-1 Abbreviations: AP-1, Activator protein 1; APE1, Apurinic/apyrimidinic endonuclease; Ref-1, Redox effector factor-1; Egr-1, Early growth response protein 1; Myb, Transcriptional activator Myb; NF-κB, Nuclear factor-κB; PAX, Paired box; P53, Tumor protein p53.

Apurinic/apyrimidinic endonuclease 1 (APE1)/Redox effector factor-1 (Ref-1) is a multifunctional protein which is ubiquitous in tissues [75]. It is involved in base excision repair of DNA damage and functions as a regulator in reductive activation of many transcription factors [74, 75]. The redox status of reactive cysteine residues, control the transcriptional activity of APE1/Ref-1 itself [74]. The C- and N- terminal region of APE1/Ref-1 are responsible for DNA repair and the redox activity, respectively (Figure 6) [74]. In base excision repair pathways, APE1/Ref-1 behaves as an apurinic/apyrimidinic endonuclease or as a 3’-phosphodiesterase and interacts with other DNA repair-related proteins [74, 75]. It maintains genomic stability in normal cells. However, the base excision repair pathway also facilitates cancer cell survival, which drives cancer growth [74, 75]. As a co-activator of transcription factors, APE1/Ref-1 controls the redox status of many transcription factors, including AP-1, NF- κB, p53, HIF-1, STAT3, Myb, PEBP2, HLF, NF-Y, Egr-1, PAX proteins, CBP/p300 and others [74, 75]. Through the activation of these transcription factors, APE1/Ref-1 is involved in cancer cell survival, proliferation, angiogenesis and apoptosis.

### Small mothers against decapentaplegic (Smad) proteins

There are 8 Smad proteins in humans. They are categorized into 3 subfamilies including receptor-regulated Smad (R-Smad) and common-partner Smad (Co-Smad) as well as inhibitory Smad (I-Smad). The R-Smad, with Smad1, Smad2, Smad3, Smad5 and Smad9, are the substrates of the receptors of the transforming growth factor-β (TGF-β) family members. Smad4 is the only Co-Smad identified in human and serves as a common partner of R-Smad. It can interact with R-Smad forming functional transcriptional complexes. The TGF-β/Smad pathway takes part in a wide range of cellular activities, such as cell growth and apoptosis. The I-Smad subfamily members Smad6 and Smad7 function in negative regulation of the TGF-β/Smad pathway. Furthermore, ROS can function as a regulator of the TGF-β/Smad pathways. It has been shown that ROS are involved in the crosstalk between the p53 and TGF-β/Smad pathways [58]. Different Smad proteins members act differently in cancer cells. Even the same Smad protein can play a dual role in cancer development [76]. Smad2, which is regarded as a suppressor of cancer, has been observed inhibited in various types of cancer [76]. Smad7 has been reported to act as a pro-carcinogenic factor by sustaining colon cancer survival. However, the over-expression of Smad7 leads to an inhibition of metastasis [76].

### Other transcription factors

For less prominent transcription factors we refer interested readers to other reviews. Yes-associated protein 1, specificity protein 1, aryl hydrocarbon receptor, early growth response protein 1, Glucocorticoid receptor, paired box 5 and 8, transcription termination factor 1 and upstream stimulatory factor 1 are described in the review of Brigelius-Flohe and Flohe [6], E26 transformation-specific-1 in the review of Verschoor et al. [16] and nuclear factor of activated T-cells as well as transcriptional activator Myb in the review of Bansal and Kaushal [37].

## DISCUSSION

### Summarizing overview on reduction-oxidation pathways

The oxidative stress theory highlights the disruption of redox signaling caused by excessive oxidants. Intracellular ROS/RNS generating organelles are mitochondria, endoplasmic reticulum and peroxisomes. In addition several enzymes generate ROS/RNS, with NOX, COX, LOX and NOS as the most prominent enzymes. Of the reactive oxidants, highly reactive hydroxyl radical and superoxide anion can react with DNA exerting harmful effects on DNA bases and structure leading to mutations. ROS/RNS also switch “on” or “off” protein activity by mediating oxidation of certain reactive amino acid residues in proteins, such as cysteine and tyrosine residues. Oxidants induce redox modifications of protein kinases and phosphatases, which subsequently play roles in the regulation of transcription factors. The aberrant activation of transcription factors results in downregulated expression of target genes related to cellular transformation, cancer cell survival and proliferation, as well as cancer angiogenesis and invasion. Oxidants induce redox modifications of protein kinases and phosphatases, which subsequently activates transcription factors. The aberrant activation of transcription factors results in the deregulation of the target gene expression. However, most of the described pathways can exert both pro- and anti- proliferative effects, depending on specific conditions.

### Opportunities for cancer prevention

Cancer prevention strategies usually aim to decrease exposure to known cancer risk factors, such as smoking, and to increase exposure to preventive factors, such as foods with anti-proliferative effects. The main risk factor for cancer, smoking, in part also acts as a carcinogen by activating oxidative pathways [79]. Therefore, smoking cessation is also a successful cancer prevention target because cessation can help to regain redox balance. Preventive agents can be found in anti-oxidative ingredients of foods and there are interesting results from investigations about actions of food ingredients on redox signaling pathways.

### Targeting mitochondria and ROS/RNS generating enzymes

Research has focused on dietary phytochemicals, which act as antioxidants in mitochondria. For instance, quercetin is a natural flavonoid compound which can be found in many fruits and vegetables. A study in Caco-2 cells showed that it protects complex I from inactivation, which has been proposed to increase the production of ROS [80]. As mentioned previously, NOX, iNOS and COX-2 are over-expressed in neoplasms. Curcumin is another phytochemical that has been reported to suppress the activation of these enzymes [81, 82]. In summary, foods rich in flavonoids, such as onions, and curcumin, such as “mango ginger” (curcuma amada), could be helpful and safe agents in cancer prevention.

### Targeting signal transduction cascades kinases/ phosphatases

*Myo*-inositol is a sugar alcohol, which can be found in whole grains, seeds and fruits. It has been reported to be an inhibitor of AKT in vitro and shows chemoprevention activity in smokers [83]. Another important target for chemoprevention is the RAS-RAF-MEK-ERK pathway, which is also frequently overactivated in cancers. Recently, both in vitro and in vivo studies have shown that the kinase activity of EKR1/2 is inhibited by grifolin, an isolate of the mushroom [84]. In addition, it has been reported in biological research that quercetin might have an effect on the activity of intracellular ROS/RNS generating enzymes via inhibiting MEK1 activity [85]. However, epidemiological evidence on the association of quercetin-rich food intake with cancer risk is still inconclusive [86]. Nevertheless, promising mechanisms acting via the redox signal transduction cascades kinases/ phosphatases have been identified by which ingredients of whole grains, seeds, fruits and mushrooms can contribute to cancer prevention.

### Targeting transcription factors

Sulforaphane and dithiolethiones act as inducers of Nrf2 and have been considered as cancer chemopreventive agents [87]. The major dietary sources of sulforaphane and dithiolethiones are cruciferous vegetables, such as broccoli. Other redox-sensitive transcription factors, such as NF-κB and AP-1, are also promising targets for cancer prevention. In addition, epigallocatechin gallate has been identified as a major antioxidant phenolic compound in green tea. It has been found to downregulate the activities of NF-κB and AP-1 in animal models [88]. In summary, cruciferous vegetables and green tea could be helpful for cancer prevention due to the beneficial effects on transcription factors.

### Cancer treatment opportunities

#### Targeting mitochondria and ROS/RNS generating enzymes

Since ROS lead to genomic instability and regulate protein function, intracellular oxidant sources are also considered as promising targets in cancer therapy. As the mitochondrial ETC generates more ROS in cancer cells, ETC inhibitors are one of the mitochondria-targeted therapeutic strategies. However, high levels of ROS make cancer cells more prone to apoptosis than normal cells and inhibiting the ETC may have unwanted proliferative side effects.

A different strategy is to inhibit oxidant-generating enzymes in order to prevent and treat cancer [21, 89]. In recent decades, efforts have been dedicated to the development of the inhibitors. For example, diphenylene iodonium is a small-molecule non-selective inhibitor and more inhibitors of NOX have been identified, recently [90]. However, most of them are not specific to NOX isoforms [91]. COX and LOX inhibitors, which are usually used as anti-inflammatory drugs, also show potential in cancer prevention or treatment [92, 93]. The COX-2 selective inhibitor Celecoxib is currently being tested in trials against lung cancer [94]. In terms of NOS, both inducers and inhibitors of NOS could be agents in cancer treatment depending on the context [26]. To be specific, fenretinide can induce iNOS-derived NO production leading to breast cancer cell death [26]. In contrast, L-NAME, an inhibitor of NOS, has been shown to reduce the production of vascular endothelial growth factor in ovarian cancer cells [95].

#### Targeting signal transduction cascades kinases/ phosphatases

This systematic review also provides an overview on the redox dependent signal transduction pathways in cancer development. Blocking components of signal transduction are a further major therapeutic strategy for developing cancer treatments. Inhibitors are currently being developed that target kinases or phosphatases of signaling pathways [32, 38, 39, 42]. Epidermal growth factor receptors, a subfamily of RTKs, are targeted by inhibitors and monoclonal antibodies which may prevent proliferation and angiogenesis in multiple cancer types, including breast cancer, colorectal cancer and lung cancer [38]. Furthermore, NRTK inhibitors are being developed for the treatment of cancer. For example, an inhibitor of Abl fusion protein has been tested for the treatment of chronic myelogenous leukemia [39]. The RAS-RAF-MEK-ERK pathway, one of the extensively studied MAPK pathways, is also a target of novel cancer therapies which focus’ on blocking this pathway via small-molecule inhibitors [96]. Furthermore, the first clinical trials have shown that inhibitors of the PI3K pathway components could be effective in various cancers [97].

#### Targeting transcription factors

Misregulation of transcription factors is a common cause of many diseases including cancer [98]. Some hyperactivated transcription factors in cancer cells have been linked with chemotherapy and radiotherapy resistance up to treatment failure [99]. Therefore, inhibition of transcription factors may be a useful strategy to increase the effectiveness of cancer therapies. For example, inhibitors are being developed to block STATs which are persistently activated in a wide range of cancers [100]. However, some transcription factors are commonly downregulated in cancer cells, mostly because of genetic aberrations [101]. Therefore, the therapeutic strategy here is to elevate the function of transcription factors or to stabilize genes encoding transcription factors. As an outstanding example, TP53 is often found mutated leading to downregulation of p53, which suppresses tumor growth and progression [58]. Nutlins, an inhibitor of a p53 blocker, has been shown to suppress tumor growth in preclinical studies by induction of p53-mediated apoptosis [101].

### Further research needs

The research area of redox signaling pathways involved in cancer development and progress is very active at the moment and identification of promising new drug targets as well as novel drug developments can be expected from future research. Although components of the redox signaling pathways are promising targets for cancer therapy and prevention, we still have a long way to go to put strategy into practice. Some challenges keep drug development research from advancing.

Firstly, redox signaling pathways concerned herein are complex and function differently under different conditions and in different cell types. Further basic research is needed to investigate mechanisms of redox signaling function in normal and cancer contexts.

Second, because of the high complexity of redox signaling pathways, manipulating them can have multiple actions that may result in severe adverse drug reactions. Clinical trials conducted by the pharmaceutical industry are therefore at high risk of failure because novel drugs may have an unfavorable benefit-risk ratio. However, this should not discourage drug development in the area of redox signaling pathways because it includes promising drug targets involved in both cancer development and progression. Further early stage clinical trials are required to assess the benefit-risk ratio of recently developed drugs and to evaluate novel drugs.

## CONCLUSIONS

In summary, this systematic review summarizes the 20 most frequently described redox-regulation pathways, which play a role in cancer formation, including ROS/RNS-generating organelles and enzymes, kinases/phosphatases in signal transduction cascades and transcription factors. The dysfunction of ROS/RNS-generating organelles and enzymes leads to ROS/RNS over-production, which induce oxidative stress. The kinases and phosphatases can be activated or inhibited by ROS/RNS and subsequently regulate the expression of transcription factors. However, whether ROS/RNS promote or suppress cancer development depends on cell context. Nevertheless, the area of redox signaling pathways includes promising drug targets involved in both cancer development and progression and further basic and clinical research is needed.

## MATERIALS AND METHODS

### Search strategy

A systematic search for literature reviews was performed in the electronic databases ISI Web of Knowledge (Thomson Scientific Technical Support, New York) and MEDLINE (Ovid Technologies, New York). Various combinations and phrases of the search terms “neoplasms”, “oxidative stress”, “free radicals” and “signal transduction” were used to find relevant publications published between January 1st, 2005 and October 20th, 2015 (Appendix A). Literature from the last ten years was considered comprehensive enough because the main discoveries were made in this time, and older, still up to date discoveries are still reviewed in more recent high-quality reviews on the topic, which we included in the systematic review. Furthermore, we filtered out publications not related to humans and other publication types than reviews. The search strategy in PubMed is shown in Appendix A. Reference lists of review articles which did not meet any exclusion criterion were checked in order to identify further articles potentially eligible for inclusion. In addition, a specific search for each redox pathway mentioned in the so far identified reviews was conducted by combining a search review for the pathway with the phases for the term neoplasms. The EndNote X7.4 software (Thompson Reuters, New York) was used throughout the literature reviews search and selection process.

### Eligibility criteria

After eliminating duplicate publications, we reviewed each title and abstract of the remaining to determine whether the article was possibly relevant for the topic of the systematic review. Papers were excluded if they (1) were an original article, (2) were not related to humans, (3) did not mention any pathway for the development of cancer, (4) described a pathway in which free radicals and redox processes were not involved, or (5) described external sources of oxidative or reductive mechanisms as the main topic (e.g. nutrients, therapeutics and toxic substances). At the stage of full-text selection, the same selection criteria were applied.

### Appraisal of the literature and extraction of information

Each included review was assessed by using a self-developed information quality score by subjectively rating the comprehensiveness and quality of information in 4 domains: Description of the redox regulation (0-3 points), description of the mechanism(s) leading to cancer (0-3 points), description of the regulator(s) of the pathway(s) (0-2 points) and description of the interaction of the main pathway of interest with other pathways (0-2 points). The maximum possible score is 10 points and reviews with a score of 6 or higher were finally included in the present review. Reviews with lower information quality scores, not being used for the present review are shown in Appendix B.

The pathways identified in the reviews and the frequencies of their appearance in the included reviews were counted. In order to focus on the most prominent pathways, we review only those pathways described 5 times or more. For readers with interest in less frequently reviewed pathways, we cite the review articles with information about these pathways.

## SUPPLEMENTARY MATERIALS TABLE


