# Supplementary material for: Reduction–oxidation pathways involved in cancer development: a systematic review of literature reviews
Source: Oncotarget. 2017 Apr 16;8(31):51888–906. doi: 10.18632/oncotarget.17128 (PMC5584299; doi:10.18632/oncotarget.17128)
Supplement: Supplementary file 1 [file oncotarget-08-51888-s001.pdf]

Reduction–oxidation pathways involved in cancer development: a systematic review of literature reviews

Supplementray Materials

Appendix

Table of contents

Appendix A - Literature search.....1

Appendix B - Excluded reviews.....2

Appendix C - Included reviews.....8

Appendix A – Literature search

Medline literature search:

Steps	Search field	Search string	Hits
#1	MeSH Terms	neoplasms	2,765,954
#2	MeSH Terms	oxidative stress	90,523
#3	MeSH Terms	free radical	244,301
#4	MeSH Terms	signal transduction	433,482
#5		#2 OR #3	301,990
#6		#1 AND #4 AND #5	2,662
#7	Filter	Review	2,089,688
#8	Filter	Humans	15,665,093
#9		#6 AND #7 AND #8 AND #9	505
#10	Date - MeSH	“2005/01/01” to “2015/10/22”	9,379,389
#11		#9 AND #10	406

Appendix B – Excluded reviews with low information quality score

1. Agani, F. and B.H. Jiang, *Oxygen-independent regulation of HIF-1: novel involvement of PI3K/AKT/mTOR pathway in cancer.* Curr Cancer Drug Targets, 2013. **13**(3): p. 245-51.
2. Baird, L. and A.T. Dinkova-Kostova, *The cytoprotective role of the Keap1-Nrf2 pathway.* Archives of Toxicology, 2011. **85**(4): p. 241-272.
3. Bauer, A.K., T. Hill, III, and C.-M. Alexander, *The Involvement of NRF2 in Lung Cancer.* Oxidative Medicine and Cellular Longevity, 2013.
4. Bauer, G., *Targeting extracellular ROS signaling of tumor cells.* Anticancer Res, 2014. **34**(4): p. 1467-82.
5. Bernard, D. and D. Vindrieux, *PLA2R1: Expression and function in cancer.* Biochimica Et Biophysica Acta-Reviews on Cancer, 2014. **1846**(1): p. 40-44.
6. Blanchetot, C. and J. Boonstra, *The ROS-NOX connection in cancer and angiogenesis.* Crit Rev Eukaryot Gene Expr, 2008. **18**(1): p. 35-45.
7. Block, K. and Y. Gorin, *Aiding and abetting roles of NOX oxidases in cellular transformation.* Nat Rev Cancer, 2012. **12**(9): p. 627-37.
8. Brierley, D.J. and S.A. Martin, *Oxidative Stress and the DNA Mismatch Repair Pathway.* Antioxidants & Redox Signaling, 2013. **18**(18): p. 2420-2428.
9. Bubici, C., et al., *The NF-kappa B-mediated control of ROS and JNK signaling.* Histology and Histopathology, 2006. **21**(1): p. 69-80.
10. Budanov, A.V., *Stress-Responsive Sestrins Link p53 with Redox Regulation and Mammalian Target of Rapamycin Signaling.* Antioxidants & Redox Signaling, 2011. **15**(6): p. 1679-1690.
11. Chen, F., K. Beezhold, and V. Castranova, *Tumor promoting or tumor suppressing of NF-kappa B, a matter of cell context dependency.* Int Rev Immunol, 2008. **27**(4): p. 183-204.
12. Cheng, H., et al., *Nitric oxide in cancer metastasis.* Cancer Lett, 2014. **353**(1): p. 1-7.
13. Chowdhury, I., W.E. Thompson, and K. Thomas, *Prohibitins Role in Cellular Survival Through Ras-Raf-MEK-Erk Pathway.* Journal of Cellular Physiology, 2014. **229**(8): p. 998-1004.
14. Clerkin, J.S., et al., *Mechanisms of ROS modulated cell survival during carcinogenesis.* Cancer Lett, 2008. **266**(1): p. 30-6.
15. Cosentino-Gomes, D., N. Rocco-Machado, and J.R. Meyer-Fernandes, *Cell signaling through protein kinase C oxidation and activation.* Int J Mol Sci, 2012. **13**(9): p. 10697-721.
16. Costa, A., A. Scholer-Dahirel, and F. Mechta-Grigoriou, *The role of reactive oxygen species and metabolism on cancer cells and their microenvironment.* Semin Cancer Biol, 2014. **25**: p. 23-32.
17. Diaz, B. and S.A. Courtneidge, *Redox signaling at invasive microdomains in cancer cells.* Free Radic Biol Med, 2012. **52**(2): p. 247-56.
18. Draganescu, C., et al., *Unravelling the connection between metabolism, cell growth and cancer.* Medecine Nucleaire-Imagerie Fonctionnelle et Metabolique, 2015. **39**(1): p. 59-63.
19. Essick, E.E. and F. Sam, *Oxidative stress and autophagy in cardiac disease, neurological disorders, aging and cancer.* Oxidative Medicine and Cellular Longevity, 2010. **3**(3): p. 168-177.
20. Felty, Q., *Redox sensitive Pyk2 as a target for therapeutics in breast cancer.* Front Biosci (Landmark Ed), 2011. **16**: p. 568-77.
21. Fogg, V.C., N.J. Lanning, and J.P. Mackeigan, *Mitochondria in cancer: at the crossroads of life and death.* Chin J Cancer, 2011. **30**(8): p. 526-39.

22. Fruehauf, J.P. and F.L. Meyskens, Jr., *Reactive oxygen species: a breath of life or death?* Clin Cancer Res, 2007. **13**(3): p. 789-94.

23. Giles, G.I., *The redox regulation of thiol dependent signaling pathways in cancer.* Curr Pharm Des, 2006. **12**(34): p. 4427-43.

24. Giudice, A. and M. Montella, *Activation of the Nrf2-ARE signaling pathway: a promising strategy in cancer prevention.* Bioessays, 2006. **28**(2): p. 169-81.

25. Grant, G.E., J. Rokach, and W.S. Powell, *5-Oxo-ETE and the OXE receptor.* Prostaglandins Other Lipid Mediat, 2009. **89**(3-4): p. 98-104.

26. Greer, E.L. and A. Brunet, *FOXO transcription factors at the interface between longevity and tumor suppression.* Oncogene, 2005. **24**(50): p. 7410-7425.

27. Grek, C.L. and K.D. Tew, *Redox metabolism and malignancy.* Curr Opin Pharmacol, 2010. **10**(4): p. 362-8.

28. Grimm, E.A., A.G. Sikora, and S. Ekmekcioglu, *Molecular pathways: inflammation-associated nitric-oxide production as a cancer-supporting redox mechanism and a potential therapeutic target.* Clin Cancer Res, 2013. **19**(20): p. 5557-63.

29. Gruening, N.-M., H. Lehrach, and M. Ralser, *Regulatory crosstalk of the metabolic network.* Trends in Biochemical Sciences, 2010. **35**(4): p. 220-227.

30. Gueron, G., A. De Siervi, and E. Vazquez, *Advanced prostate cancer: reinforcing the strings between inflammation and the metastatic behavior.* Prostate Cancer Prostatic Dis, 2012. **15**(3): p. 213-21.

31. Hayes, P. and U.G. Knaus, *Balancing reactive oxygen species in the epigenome: NADPH oxidases as target and perpetrator.* Antioxid Redox Signal, 2013. **18**(15): p. 1937-45.

32. Hess, J., P. Angel, and M. Schorpp-Kistner, *AP-1 subunits: quarrel and harmony among siblings.* J Cell Sci, 2004. **117**(Pt 25): p. 5965-73.

33. Ishdorj, G., L. Li, and S.B. Gibson, *Regulation of autophagy in hematological malignancies: role of reactive oxygen species.* Leuk Lymphoma, 2012. **53**(1): p. 26-33.

34. Jaganjac, M., *Possible involvement of granulocyte oxidative burst in Nrf2 signaling in cancer.* Indian Journal of Medical Research, 2010. **131**(5): p. 609-616.

35. Jaramillo, M.C. and D.D. Zhang, *The emerging role of the Nrf2-Keap1 signaling pathway in cancer.* Genes & Development, 2013. **27**(20): p. 2179-2191.

36. Jiang, P., W. Du, and M. Wu, *Regulation of the pentose phosphate pathway in cancer.* Protein & Cell, 2014. **5**(8): p. 592-602.

37. Kamata, T., *Roles of Nox1 and other Nox isoforms in cancer development.* Cancer Sci, 2009. **100**(8): p. 1382-8.

38. Kang, J.-H., *Protein Kinase C (PKC) Isozymes and Cancer.* New Journal of Science, 2014. **2014**(2014): p. 36.

39. Katoh, Y. and M. Katoh, *FGFR2-related pathogenesis and FGFR2-targeted therapeutics.* International Journal of Molecular Medicine, 2009. **23**(3): p. 307-311.

40. Kelley, M.R., M.M. Georgiadis, and M.L. Fishel, *APE1/Ref-1 role in redox signaling: translational applications of targeting the redox function of the DNA repair/redox protein APE1/Ref-1.* Curr Mol Pharmacol, 2012. **5**(1): p. 36-53.

41. Kensler, T.W., N. Wakabayash, and S. Biswal, *Cell survival responses to environmental stresses via the Keap1-Nrf2-ARE pathway*, in *Annual Review of Pharmacology and Toxicology*. 2007. p. 89-116.

42. Lau, A., et al., *Dual roles of Nrf2 in cancer.* Pharmacological Research, 2008. **58**(5-6): p. 262-270.

43. Lebiedzinska-Arciszewska, M., et al., *The interplay between p66Shc, reactive oxygen species and cancer cell metabolism.* Eur J Clin Invest, 2015. **45 Suppl 1**: p. 25-31.

44. Leon, L., J.F. Jeannin, and A. Bettaieb, *Post-translational modifications induced by nitric oxide (NO): implication in cancer cells apoptosis.* Nitric Oxide, 2008. **19**(2): p. 77-83.

45. Li, L., G. Ishdorj, and S.B. Gibson, *Reactive oxygen species regulation of autophagy in cancer: implications for cancer treatment.* Free Radic Biol Med, 2012. **53**(7): p. 1399-410.

46. Liontos, M., et al., *The tumor suppressor gene ARF as a sensor of oxidative stress.* Curr Mol Med, 2012. **12**(6): p. 704-15.

47. Liou, G.Y. and P. Storz, *Reactive oxygen species in cancer.* Free Radic Res, 2010. **44**(5): p. 479-96.

48. Liu, B., L. Qu, and S. Yan, *Cyclooxygenase-2 promotes tumor growth and suppresses tumor immunity.* Cancer Cell Int, 2015. **15**: p. 106.

49. Liu, H., et al., *An unconventional role of BMP-Smad1 signaling in DNA damage response: a mechanism for tumor suppression.* J Cell Biochem, 2014. **115**(3): p. 450-6.

50. Liu, W., et al., *Targeted genes and interacting proteins of hypoxia inducible factor-1.* Int J Biochem Mol Biol, 2012. **3**(2): p. 165-78.

51. Lopez-Lazaro, M., *Role of Oxygen in Cancer: Looking Beyond Hypoxia.* Anti-Cancer Agents in Medicinal Chemistry, 2009. **9**(5): p. 517-525.

52. Low, I.C.C., J. Kang, and S. Pervaiz, *Bcl-2: A Prime Regulator of Mitochondrial Redox Metabolism in Cancer Cells.* Antioxidants & Redox Signaling, 2011. **15**(12): p. 2975-2987.

53. Lu, J., et al., *Oxidative Stress and Lectin-Like Ox-LDL-Receptor LOX-1 in Atherogenesis and Tumorigenesis.* Antioxidants & Redox Signaling, 2011. **15**(8): p. 2301-2333.

54. Lu, J., L.K. Sharma, and Y. Bai, *Implications of mitochondrial DNA mutations and mitochondrial dysfunction in tumorigenesis.* Cell Res, 2009. **19**(7): p. 802-15.

55. Luo, J.L., H. Kamata, and M. Karin, *The anti-death machinery in IKK/NF-kappa B signaling.* Journal of Clinical Immunology, 2005. **25**(6): p. 541-550.

56. Ma, Z. and K. Vosseller, *Cancer Metabolism and Elevated O-GlcNAc in Oncogenic Signaling.* Journal of Biological Chemistry, 2014. **289**(50): p. 34457-34465.

57. Macleod, K.F., *The role of the RB tumour suppressor pathway in oxidative stress responses in the haematopoietic system.* Nat Rev Cancer, 2008. **8**(10): p. 769-81.

58. Maiese, K., et al., *Clever cancer strategies with FoxO transcription factors.* Cell Cycle, 2008. **7**(24): p. 3829-3839.

59. Martinez-Outschoorn, U., F. Sotgia, and M.P. Lisanti, *Tumor microenvironment and metabolic synergy in breast cancers: critical importance of mitochondrial fuels and function.* Semin Oncol, 2014. **41**(2): p. 195-216.

60. Martinez-Outschoorn, U.E., M.P. Lisanti, and F. Sotgia, *Catabolic cancer-associated fibroblasts transfer energy and biomass to anabolic cancer cells, fueling tumor growth.* Semin Cancer Biol, 2014. **25**: p. 47-60.

61. Martinez-Sanchez, G. and A. Giuliani, *Cellular redox status regulates hypoxia inducible factor-1 activity. Role in tumour development.* J Exp Clin Cancer Res, 2007. **26**(1): p. 39-50.

62. Mates, J.M., et al., *Oxidative stress in apoptosis and cancer: an update.* Arch Toxicol, 2012. **86**(11): p. 1649-65.

63. Maynard, S., et al., *Base excision repair of oxidative DNA damage and association with cancer and aging.* Carcinogenesis, 2009. **30**(1): p. 2-10.

64. Mehrotra, S., et al., *Oxidative Stress and Lymphocyte Persistence: Implications in Immunotherapy*, in *Advances in Cancer Research, Vol 102*, G.F.V. Woude and G. Klein, Editors. 2009. p. 197-227.

65. Mellier, G. and S. Pervaiz, *The three Rs along the TRAIL: resistance, re-sensitization and reactive oxygen species (ROS).* Free Radic Res, 2012. **46**(8): p. 996-1003.

66. Menna, C., et al., *Lipoxygenase inhibitors for cancer prevention: promises and risks.* Curr Pharm Des, 2010. **16**(6): p. 725-33.

67. Miller, T.W., J.S. Isenberg, and D.D. Roberts, *Molecular regulation of tumor angiogenesis and perfusion via redox signaling.* Chem Rev, 2009. **109**(7): p. 3099-124.

68. Moscat, J. and M.T. Diaz-Meco, *p62: a versatile multitasker takes on cancer.* Trends Biochem Sci, 2012. **37**(6): p. 230-6.

69. Nezis, I.P. and H. Stenmark, *p62 at the interface of autophagy, oxidative stress signaling, and cancer.* Antioxid Redox Signal, 2012. **17**(5): p. 786-93.

70. Nogueira, V. and N. Hay, *Molecular pathways: reactive oxygen species homeostasis in cancer cells and implications for cancer therapy.* Clin Cancer Res, 2013. **19**(16): p. 4309-14.

71. North, S., M. Moenner, and A. Bikfalvi, *Recent developments in the regulation of the angiogenic switch by cellular stress factors in tumors.* Cancer Lett, 2005. **218**(1): p. 1-14.

72. Nourazarian, A.R., P. Kangari, and A. Salmaninejad, *Roles of Oxidative Stress in the Development and Progression of Breast Cancer.* Asian Pacific Journal of Cancer Prevention, 2014. **15**(12): p. 4745-4751.

73. Ostrakhovitch, E.A., *Redox Environment and its Meaning for Breast Cancer Cells Fate.* Current Cancer Drug Targets, 2011. **11**(4): p. 479-495.

74. Pan, J.S., M.Z. Hong, and J.L. Ren, *Reactive oxygen species: a double-edged sword in oncogenesis.* World J Gastroenterol, 2009. **15**(14): p. 1702-7.

75. Pascucci, B., et al., *Role of nucleotide excision repair proteins in oxidative DNA damage repair: an updating.* Biochemistry-Moscow, 2011. **76**(1): p. 4-15.

76. Patra, K.C. and N. Hay, *The pentose phosphate pathway and cancer.* Trends in Biochemical Sciences, 2014. **39**(8): p. 347-354.

77. Perl, A., et al., *Oxidative stress, inflammation and carcinogenesis are controlled through the pentose phosphate pathway by transaldolase.* Trends in Molecular Medicine, 2011. **17**(7): p. 395-403.

78. Quaegebeur, A. and P. Carmeliet, *Oxygen Sensing: A Common Crossroad in Cancer and Neurodegeneration*, in *Diverse Effects of Hypoxia on Tumor Progression*, M.C. Simon, Editor. 2010. p. 71-103.

79. Rajendran, M., et al., *p66Shc-a longevity redox protein in human prostate cancer progression and metastasis.* Cancer and Metastasis Reviews, 2010. **29**(1): p. 207-222.

80. Reuter, S., et al., *Oxidative stress, inflammation, and cancer: how are they linked?* Free Radic Biol Med, 2010. **49**(11): p. 1603-16.

81. Riganti, C., et al., *The pentose phosphate pathway: An antioxidant defense and a crossroad in tumor cell fate.* Free Radical Biology and Medicine, 2012. **53**(3): p. 421-436.

82. Rojas-Rivera, D. and C. Hetz, *TMBIM protein family: ancestral regulators of cell death.* Oncogene, 2015. **34**(3): p. 269-80.

83. Roy, K., et al., *NADPH oxidases and cancer.* Clin Sci (Lond), 2015. **128**(12): p. 863-75.

84. Sardina, J.L., et al., *Reactive oxygen species: are they important for haematopoiesis?* Crit Rev Oncol Hematol, 2012. **81**(3): p. 257-74.

85. Sauer, H. and M. Wartenberg, *Reactive oxygen species as signaling molecules in cardiovascular differentiation of embryonic stem cells and tumor-induced angiogenesis.* Antioxid Redox Signal, 2005. **7**(11-12): p. 1423-34.

86. Saw, C.L.L. and A.-N.T. Kong, *Nuclear factor-erythroid 2-related factor 2 as a chemopreventive target in colorectal cancer.* Expert Opinion on Therapeutic Targets, 2011. **15**(3): p. 281-295.

87. Seki, E., D.A. Brenner, and M. Karin, *A Liver Full of JNK: Signaling in Regulation of Cell Function and Disease Pathogenesis, and Clinical Approaches.* Gastroenterology, 2012. **143**(2): p. 307-320.

88. Semenza, G.L., *Regulation of cancer cell metabolism by hypoxia-inducible factor 1.* Seminars in Cancer Biology, 2009. **19**(1): p. 12-16.

89. Sesti, F., et al., *Oxidative stress-mediated biomolecular damage and inflammation in tumorigenesis.* In Vivo, 2012. **26**(3): p. 395-402.

90. Shiota, M., A. Yokomizo, and S. Naito, *Oxidative stress and androgen receptor signaling in the development and progression of castration-resistant prostate cancer.* Free Radic Biol Med, 2011. **51**(7): p. 1320-8.

91. Singleton, D.C. and A.L. Harris, *Targeting the ATF4 pathway in cancer therapy.* Expert Opinion on Therapeutic Targets, 2012. **16**(12): p. 1189-1202.

92. Slattery, M.L. and F.A. Fitzpatrick, *Convergence of Hormones, Inflammation, and Energy-Related Factors: A Novel Pathway of Cancer Etiology.* Cancer Prevention Research, 2009. **2**(11): p. 922-930.

93. Sosa, V., et al., *Oxidative stress and cancer: An overview.* Ageing Research Reviews, 2013. **12**(1): p. 376-390.

94. Storz, P., *Reactive oxygen species in tumor progression.* Front Biosci, 2005. **10**: p. 1881-96.

95. Sullivan, R. and C.H. Graham, *Chemosensitization of cancer by nitric oxide.* Curr Pharm Des, 2008. **14**(11): p. 1113-23.

96. Susnow, N., et al., *Bcl-2 family proteins as regulators of oxidative stress.* Seminars in Cancer Biology, 2009. **19**(1): p. 42-49.

97. Svilar, D., et al., *Base Excision Repair and Lesion-Dependent Subpathways for Repair of Oxidative DNA Damage.* Antioxidants & Redox Signaling, 2011. **14**(12): p. 2491-2507.

98. Taguchi, K., H. Motohashi, and M. Yamamoto, *Molecular mechanisms of the Keap1-Nrf2 pathway in stress response and cancer evolution.* Genes Cells, 2011. **16**(2): p. 123-40.

99. Tang, S., et al., *The role of oxidized ATM in the regulation of oxidative stress-induced energy metabolism reprogramming of CAFs.* Cancer Lett, 2014. **353**(2): p. 133-44.

100. Tao, R., et al., *Regulation of MnSOD enzymatic activity by Sirt3 connects the mitochondrial acetylome signaling networks to aging and carcinogenesis.* Antioxid Redox Signal, 2014. **20**(10): p. 1646-54.

101. Tudek, B., *Base excision repair modulation as a risk factor for human cancers.* Molecular Aspects of Medicine, 2007. **28**(3-4): p. 258-275.

102. Tuncer, S. and S. Banerjee, *Eicosanoid pathway in colorectal cancer: Recent updates.* World J Gastroenterol, 2015. **21**(41): p. 11748-66.

103. Turpaev, K.T., *Keap1-Nrf2 signaling pathway: Mechanisms of regulation and role in protection of cells against toxicity caused by xenobiotics and electrophiles.* Biochemistry-Moscow, 2013. **78**(2): p. 111-126.

104. Tzivion, G., M. Dobson, and G. Ramakrishnan, *FoxO transcription factors; Regulation by AKT and 14-3-3 proteins.* Biochimica Et Biophysica Acta-Molecular Cell Research, 2011. **1813**(11): p. 1938-1945.

105. Valko, M., et al., *Free radicals, metals and antioxidants in oxidative stress-induced cancer.* Chem Biol Interact, 2006. **160**(1): p. 1-40.

106. Vandenabeele, P., et al., *The role of the kinases RIP1 and RIP3 in TNF-induced necrosis.* Sci Signal, 2010. **3**(115): p. re4.

107. Vriend, J. and R.J. Reiter, *The Keap1-Nrf2-antioxidant response element pathway: A review of its regulation by melatonin and the proteasome.* Molecular and Cellular Endocrinology, 2015. **401**(C): p. 213-220.

108. Wallace, D.C., *Mitochondria and cancer: Warburg addressed.* Cold Spring Harb Symp Quant Biol, 2005. **70**: p. 363-74.

109. Wang, Z., Y. Li, and F.H. Sarkar, *Signaling mechanism(s) of reactive oxygen species in Epithelial-Mesenchymal Transition reminiscent of cancer stem cells in tumor progression.* Curr Stem Cell Res Ther, 2010. **5**(1): p. 74-80.

110. Weinberg, F. and N.S. Chandel, *Reactive oxygen species-dependent signaling regulates cancer.* Cell Mol Life Sci, 2009. **66**(23): p. 3663-73.

111. Wu, W.S., *The signaling mechanism of ROS in tumor progression.* Cancer Metastasis Rev, 2006. **25**(4): p. 695-705.

112. Wu, W.S., J.R. Wu, and C.T. Hu, *Signal cross talks for sustained MAPK activation and cell migration: the potential role of reactive oxygen species.* Cancer Metastasis Rev, 2008. **27**(2): p. 303-14.

113. Wu, Y., et al., *Molecular mechanisms underlying chronic inflammation-associated cancers.* Cancer Lett, 2014. **345**(2): p. 164-73.

114. Yang, W., et al., *Redox regulation of cancer metastasis: molecular signaling and therapeutic opportunities.* Drug Dev Res, 2014. **75**(5): p. 331-41.

115. Yang, Y., et al., *Reactive oxygen species in cancer biology and anticancer therapy.* Curr Med Chem, 2013. **20**(30): p. 3677-92.

116. Zhang, C., et al., *Cancer may be a pathway to cell survival under persistent hypoxia and elevated ROS: A model for solid-cancer initiation and early development.* International Journal of Cancer, 2015. **136**(9): p. 2001-2011.

117. Zhang, L., Z. Yang, and Y. Liu, *GADD45 proteins: roles in cellular senescence and tumor development.* Experimental Biology and Medicine, 2014. **239**(7): p. 773-778.

118. Zhang, S., et al., *Homeostasis of redox status derived from glucose metabolic pathway could be the key to understanding the Warburg effect.* American Journal of Cancer Research, 2015. **5**(4): p. 1265-1280.

119. Zhang, Y., et al., *FoxO family members in cancer.* Cancer Biology & Therapy, 2011. **12**(4): p. 253-259.

120. Zheng, Q., J. Ye, and J. Cao, *Translational regulator eIF2 alpha in tumor.* Tumor Biology, 2014. **35**(7): p. 6255-6264.

Appendix C – Included reviews

High-quality reviews scored with 6 point or more in information quality score

No.	Authors	Year	Described pathway(s)	Redox regulation	Mechanism(s) leading to cancer	Component of pathway(s)	Interaction with other pathways	Overall score
				**Max. possible points: 3**	**Max. possible points: 3**	**Max. possible points: 2**	**Max. possible points: 2**	**Max. possible score:10**
1	Aggarwal, B.B., et al.	2006	NF-κB	1.5	1.5	1.5	2.0	6.5
2	Bansal, M. and Kaushal, N.	2014	Redox signaling	3.0	0.0	2.0	2.0	7.0
3	Barthel, A. and Klotz, L. O.	2005	PI3K	2.5	1.0	1.0	1.5	6.0
4	Bogdan, C.	2015	NOS	3.0	0.0	2.0	1.0	6.0
5	Bourgeais, J., et al.	2013	STAT	2.5	2.5	1.0	1.5	7.5
6	Brigelius-Flohe, R. and Flohe, L.	2011	Transcription factors	2.5	1.5	1.0	1.0	6.0
7	Chiarugi, P.	2005	PTP and PTK	3.0	1.0	1.0	1.5	6.5
8	Dhillon, A. S., et al.	2007	MAPK	1.5	3.0	1.5	1.0	7.0
9	Duan, C.	2016	HIF-3	2.5	2.0	0.5	1.0	6.0
10	Eferl, R. and Wagner, E. F.	2003	AP-1	1.0	3.0	1.5	1.0	6.5
11	Fukumura, D., et al.	2006	NOS	2.0	2.5	1.0	1.0	6.5
12	Furstenberger, G., et al	2006	COX and LOX	2.0	2.0	1.0	1.0	6.0
13	Galanis, A., et al.	2008	HIF-1	3.0	2.0	0.5	1.5	7.0
14	Garg, R., et al.	2014	PKC	1.0	3.0	2.0	0.5	6.5
15	Giorgi, C., et al.	2010	PKC	2.5	1.5	1.0	2.0	7.0
16	Gocek, E., et al.	2014	NRTK	0.5	2.5	2.0	1.0	6.0
17	Holmstrom, K. M. and Finkel, T.	2014	ROS/RNS sources	2.5	1.5	1.0	2.0	7.0
18	Jiang, F., et al.	2011	NOX	2.0	1.0	1.0	2.0	6.0
19	Kappelmann, M., et al.	2014	AP-1	1.0	2.5	1.5	1.0	6.0
20	Karamouzis, M. V., et al.	2007	AP-1	0.0	2.5	1.5	2.0	6.0
21	Kim, E. K. and Choi, E.-J.	2015	MAPK	1.5	2.5	1.0	1.0	6.0
22	Knab, L. M., et al.	2014	COX and LOX	1.5	2.5	1.0	1.0	6.0

No.	Authors	Year	Described pathway(s)	Redox regulation	Mechanism(s) leading to cancer	Component of pathway(s)	Interaction with other pathways	Overall score
				**Max. possible points: 3**	**Max. possible points: 3**	**Max. possible points: 2**	**Max. possible points: 2**	**Max. possible score:10**
23	Korbecki, J., et al.	2013	COX	2.5	0.5	1.5	2.0	6.5
24	Kotsinas, A., et al.	2012	p53	2.5	2.5	1.0	0.0	6.0
25	Ladelfa, M. F., et al.	2011	p53, PI3K, Wnt/β-catenin, HIF-1, MAPK	2.5	1.5	1.0	1.5	6.5
26	Landry, W. D. and Cotter, T. G.	2014	NOX	2.5	2.5	1.0	1.0	7.0
27	Leinonen, H. M., et al.	2014	Nrf2	1.5	2.0	2.0	2.0	7.5
28	Liu, W. and Phang, J. M.	2012	Proline dehydrogenase	2.5	2.0	0.5	1.5	6.5
29	MacDonald, B. T., et al.	2009	Wnt/β-catenin	2.0	2.0	2.0	1.5	7.5
30	Maillet, A. and Pervaiz, S.	2012	p53	2.5	2.5	0.5	2.0	7.5
31	Martini, M., et al.	2014	PI3Ks	1.5	2.5	1.5	1.0	6.5
32	McCubrey, J. A., et al.	2006	MAPK	2.5	1.5	1.5	1.0	6.5
33	Meitzler, J. L., et al.	2014	NOX	2.5	2.5	1.0	1.0	7.0
34	Monga, S. P.	2011	Wnt/β-catenin	1.5	2.5	1.5	1.0	6.5
35	Movafagh, S., et al.	2015	HIF-1	2.5	1.5	1.0	1.0	6.0
36	Myatt, S. S., et al.	2011	FOXO	2.5	2.5	1.0	0.5	6.5
37	Na, H. K. and Surh, Y. J.	2014	Nrf2	2.0	1.5	1.0	1.5	6.0
38	Nakanishi, A., et al.	2014	PI3K	2.0	1.5	1.5	1.0	6.0
39	Oeckinghaus, A. and Ghosh, S.	2009	NF-κB	2.5	1.5	1.5	1.0	6.5
40	Ostman, A., et al.	2006	PTP	2.0	1.5	1.0	1.5	6.0
41	Pani, G. and Galeotti, T.	2011	P53	2.5	1.5	1.0	2.0	7.0
42	Parri, M. and Chiarugi, P.	2013	PTK, PTP, P53, Nrf2, Nf-kB	2.5	2.5	1.0	1.0	7.0
43	Qin, F., et al.	2013	Protein kinase Hippo	2.0	2.0	1.5	0.5	6.0
44	Regad, T.	2015	RTKs	1.0	2.5	1.5	1.5	6.5
45	Saito-Diaz, K., et al.	2013	Wnt/β-catenin	1.5	2.0	2.0	1.0	6.5

No.	Authors	Year	Described pathway(s)	Redox regulation	Mechanism(s) leading to cancer	Component of pathway(s)	Interaction with other pathways	Overall score
				Max. possible points: 3	Max. possible points: 3	Max. possible points: 2	Max. possible points: 2	Max. possible score:10
46	Samanta, D. and Datta, P.K.	2012	Smad	0.5	2.5	2.0	1.0	6.0
47	Sarkar, F. H., et al.	2008	NF-κB	2.0	2.0	1.0	1.0	6.0
48	Shelton, P. and Jaiswal, A. K.	2013	Nrf2	2.5	1.0	0.5	2.0	6.0
49	Speed, N. and Blair, I. A.	2011	COX and LOX	2.0	2.0	1.0	1.0	6.0
50	Storz, P.	2011	FOXO	2.5	1.5	1.0	2.0	7.0
51	Tell, G., et al.	2009	APE1/Ref-1	3.0	1.0	0.5	2.0	6.5
52	Thakur, S., et al.	2014	APE1/Ref-1	1.5	1.5	1.0	2.0	6.0
53	Tokarz, P. and Blasiak, J.	2014	Mitochondria	1.5	2.5	1.0	1.0	6.0
54	Tonks, N. K.	2006	PTP	1.5	2.5	2.0	0.5	6.5
55	Vannini, F., et al.	2015	NOS	2.0	2.5	1.0	1.0	6.5
56	Verschoor, M. L., et al.	2010	Est-1	2.5	2.5	1.0	0.5	6.5
57	Vurusaner, B., et al.	2012	P53, FOXO	2.5	2.5	0.0	1.0	6.0
58	Wagner, E. F. and Nebreda, A. R.	2009	MAPK	2.0	3.0	1.0	1.0	7.0
59	Wallace, D. C.	2012	Mitochondria	2.5	2.5	0.0	1.5	6.5
60	Watroba, M., et al.	2012	FOXO	2.5	1.0	1.0	1.5	6.0
61	Xiang, M., et al.	2014	Nrf2	2.0	2.5	1.0	0.5	6.0
62	Yang, Q. and Lee, J. D.	2011	MAPK	0.5	3.0	1.5	1.0	6.0
63	Yousefi, B., et al.	2014	PI3K	1.5	2.5	1.0	1.0	6.0
64	Yu, H., et al.	2009	STAT	0.5	3.0	2.0	1.5	7.0
65	Zhao, J., et al.	2015	HIF-2	1.5	2.5	1.0	1.0	6.0

Abbreviations: AP-1, Activator protein 1; APE1/Ref-1, Apurinic/apyrimidinic endonuclease 1/Redox effector factor-1; COX, Cyclooxygenase; FOXO, Forkhead box O; HIF, Hypoxia-inducible factors; LOX, Lipoxygenase; MAPK, Mitogen-activated protein kinase; NF-κB,,Nuclear factor-κB; NOS, Nitric oxide synthase; NOX, NADPH oxidase; Nrf2, Nuclear factor E2-related factor 2; P53, Tumor protein p53; PI3K, Phosphoinositide 3-kinase; PKC, Protein kinase C; PTK, Protein tyrosine kinase; PTP, protein tyrosine phosphatase; ROS, Reactive oxygen species; Smad , Small mothers against decapentaplegic; STAT, Signal transducer and activator of transcription.
